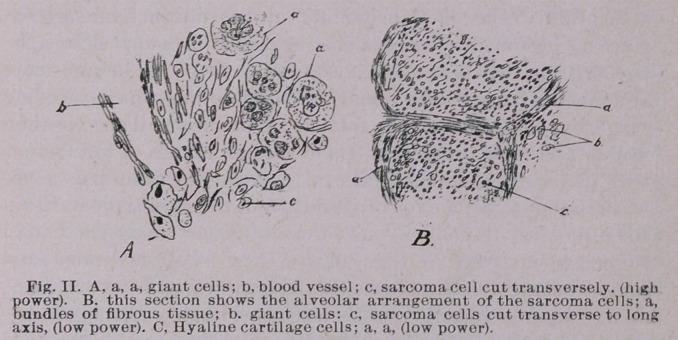# A Case of Chondro-Sarcoma of the Chest

**Published:** 1897-02

**Authors:** Wilson T. Davidson

**Affiliations:** Belton, Texas, Interne John Sealy Hospital, Galveston, Texas


### TEXAS MEDICAL JOURNAL.

ESTABLISHED JULY, 1885.

PUBLISHED MONTHLY.—SUBSCRIPTION $2.00 A YEAR.

VOL. XII. AUSTIN, FEBRUARY, 1897. NO. 8.

### Original Contributions.

For the Texas Medical Journal.

### A CASE OF CHONDRO-SARCOMA OF THE CHEST.

BY WILSON T. DAVIDSON, B. SC., M. D., BELTON, TEXAS,
Interne John Sealy Hospital, Galveston, Texas.

HISTORY.—Mrs. L. W.; age, forty-five; married. Was admitted to John Sealy Hospital, February 14, 1896. There was no history of any tumor growth in the family. She has had six children, the eldest being twenty, and the youngest six years of age. *Previous personal history.*—The patient's health has always been good; gives no history whatever of syphilis, gout, rheumatism, or any other constitutional taint; habits have always been good, and the patient has always led an active life.

*Present illness.*—One year ago from last August a small subcutaneous nodule about the size of the end of one's finger was noticed two inches below and a little to the right of the left nipple. It felt more like cartilage than anything else. There was no dimpling of skin; no pain. The growth was observed to increase in size, the increase becoming more and more rapid as it grew older. In January, 1895, it was about the size of a large pecan; and last summer (August), that of a hen's egg. From this time on the tumor grew very fast, and become *painful.* This pain was of a deep boring character, with occasionally a prickling sensation towards its sternal side. The pain was constant, and did not vary in its intensity at any time during the day. From the time in August, when first noticed, up to the present time, the pain has grown more and more intense, so much so, that for the last month or so, the patient has been kept awake at night.

*Physical examination.*—The patient is fairly well nourished. She has noticed no digestive disturbance since the tumor appeared; no axillary enlargement. Has fallen off about ten pounds in the last few months. The lungs and heart normal. Examination of the urine was negative. *Inspection* reveals a tumor about the size of one's fist, on the left side of the chest, having a nodular appearance. There is, in fact, a distinctly protuberant nodule extending out from the surface. It occupies the sternal portion of the fourth, fifth, sixth and seventh ribs, and their costal cartilages. The bulk of the tumor is below, and to the inner side of the nipple. *Palpation.*—The mass is hard, firmly adherent to the ribs. In moving the tumor, the ribs move with it, just as though they were a part of the same. No points of fluctuation. Indistinct outline at edges. The breast can be distinctly outlined above the tumor as separate and distinct from it. The skin is not adherent to growth. No tenderness complained of upon palpation.

*Diagnosis.*—Of the various new growths that could occur in this situation, let us consider them one by one, and show why they were excluded. *Chondromata.*—The fact that chondromata are painless, grow slowly, and are distinctly encapsulated, would warrant us in putting them aside. *Osteomata.*—These tumors grow very slowly, and are free from pain. *Lipomata.*—These are encapsulated, of slow growth, and without pain; hence, excluded also. *Fibromata.*—These, like myomata, are to be excluded, from the fact that they are both without pain, and do not grow so rapidly as the tumor under consideration.

It will be remembered that there is a good deal of pain associated with this growth; that this pain is constant, and that it has increased in severity very rapidly. Again, the increase in size of this tumor has been rapid. As time passed by, it has increased in size somewhat after the manner of a geometrical progression. These facts point distinctly to malignancy; and as there is no glandular tissue that would give rise to a cancer in this position, sarcoma is decided upon as more nearly fulfilling all the symptoms than any other tumor. A pure sarcoma is soft; it is made up of embryonal connective tissue cells, and a small amount of fibrous tissue. It will be remembered, this tumor is *hard*; inasmuch, therefore, as it is growing from cartilage, the inference is made that this increase in consistency is brought about by cartilagenous tissue; and as it would take considerable cartilage to give such firmness as this tumor presents, we are warranted in concluding that the cartilage predominates. So the diagnosis of *chondro-sarcoma* is made, and that the proportion of cartilage is greater than that of sarcomatous tissue.

*Operation.*—The patient was prepared for operation as follows: At 8 o'clock the night preceding the operation a saline, consisting of half an ounce magnesium sulphate was given, and the part shaved and thoroughly washed with sterile water and bichloride solution, and wrapped in towels that had been soaked in bichloride solution of the strength of 1:1000. On the following morning at 5 o'clock, she was allowed a cup of tea, but no further nourishment before the operation; an enema of two pints of soap and water and a bath were administered. The operation began at 9 o'clock a. m., February 17th. Immediately preceding the operation, the part was shaved again, washed in sterile water, field of operation colored with a solution of permanganate of potassium, and this latter taken off with an oxalic acid solution, and again washed with warm sterile water. Prof. J. E. Thompson did the operation, assisted by Prof. William Keiller, Dr. Louis Magnenat, and the writer. An incision was made through the skin, beginning at the lower border of the sternal articulation of the third rib, and extending in a semilunar direction downwards and outwards to the upper border of the sixth rib; thence outwards and upwards to a point three inches to the left of the nipple. (See Fig. 1.) Immediately below the skin the tumor appeared, perfectly free from breast tissue. A few blood vessels were clamped, and the flap retracted, carrying with it the mammary gland. The tumor was now cleared of the tissues around it. Immediately to the left side of the tumor, the third and fourth ribs were exposed, and freed from their periosteum, and then cut directly through with bone forceps. These two ribs were again cut about three-fourths to one inch more to median line, thus allowing a small piece of each of these ribs and the parietal pleura to be removed. Now the lung was exposed, and could be seen expanding with each inspiration, but not yet collapsed. The index and middle fingers were then passed into the pleural cavity and used as directors, and with a stout pair of curved scissors, the cartilagenous portion of the third rib was cut loose, then the sternal attachments of the fourth and fifth, and finally the cartilage of the sixth rib was severed. The pleura was included. Thus a hole about three inches in diameter was made, and the lung seen expanding under our eyes, and the heart beating away slowly, labored, from the chloroform anesthesia. The lung became more and more collapsed from exposure. There was some bleeding from the intercostals and internal mammary. This bleeding was stopped by pressure. The skin flap was brought together with interrupted sutures of catgut. A plug of iodoform gauze was allowed to protrude through the outer part of the incision for drainage. Iodoform powder was dusted along the line of suture, a small strip of gauze and sterile cotton applied, and these were held firmly in place by a many-tailed bandage.

*History Subsequent to the Operation.*—The operation was finished at 10:15 a. m. Strychnine, $\frac{1}{30}$ of a grain had been given twice during the operation. The patient was brought down from the operating room, covered well, and hot bottles applied. Strychnine, $\frac{1}{30}$, and morphine, $\frac{1}{8}$ of a grain, were given immediately after the patient was put to bed. Temperature, at 1:50, was 95.6° F.; respiration, 32, and the pulse, 66, very weak. At 11:30 temperature was 96° F.; respiration, 32, and the pulse still 66, and continued very weak. At 1:30 p. m. the temperature was 96°, and the pulse and respiration, 71 and 24, respectively. The pulse was very good. At 5:30 p. m. the temperature was 98°, the pulse 84, and the respiration 22. The patient complained now of dyspnœa and pain in precordial region and left shoulder; very thirsty. A small quantity of ice water was given. At 4:20 p. m. strychnine, $\frac{1}{30}$, and morphine, $\frac{1}{8}$ of a grain, were given hypodermically. Patient vomited slightly at 6 p. m., matters of light green color. The urine was voided voluntarily at 6:30 p. m. Vomited again at 6:40 p. m. At 2 o'clock in the following morning a pain was complained of in the left diaphragmatic region. This was probably a slight pleurisy. Morphine, $\frac{1}{8}$ of a grain, was administered for the pain, and the patient slept well at intervals during the night. The following morning she seemed in a good condition. The temperature was 99.5°, pulse, 98, and the respiration 24. The general appearance was good. The patient was now put on nourishment, in the way of peptonized milk, beef-tea, and whisky. The temperature, at 8 p. m., was 100° F. The temperature was slight for about one week. The bowels were moved by enemata and sulphate of magnesium. After this our patient's appetite continued to improve, and she was given additional nourishment in the way of

Fig. II. A, a, a, giant cells; b, blood vessel; c, sarcoma cell cut transversely. (high power). B. this section shows the alveolar arrangement of the sarcoma cells; a, bundles of fibrous tissue; b. giant cells: c, sarcoma cells cut transverse to long axis, (low power). C, Hyaline cartilage cells; a, a, (low power).

beef, toast and eggs. The gauze was removed from the wound on the third day, and fresh dressing applied. The wound continued to heal, and absolute union by first intention was secured. Upon examination the chest, a few days after the operation, showed a considerable tympanitic note for the left lung, and a "sea-sawing" motion by the respiratory act. Both these symptoms became less marked as convalescence progressed. Inasmuch as the air in the pleural cavity was kept from becoming septic, no harm could result from that source. The apex beat of the heart was nicely shown. Convalescence was rapid, and the patient left the hospital, March 3rd, with a good appetite, strong enough to sit up all day, and very much pleased with the result of the operation.

*Examination of the Tumor.*—To the touch the tumor removed is firm; cuts with some resistance. On the under surface hard, smooth nodules of cartilage may be felt. Upon microscopical examination, the tumor is seen to be made up of large spindle cells arranged in alveoli, with considerable cartilage in a hyaline matrix, and giant cells scattered here and there in groups. (See Fig. II, A, B and C.)

*Dissemination and Recurrence.*—Referring again to the histological structure of the tumor, it will be noticed that the vessels have walls which are made up almost entirely of sarcoma cells. (See Fig. II, A, B.) This being so, it is easy to see how a few cells may become detached and thrown into the venous circulation. In larger veins it frequently happens that the sarcomatous tissue makes its way through their walls. Later on it is possible for these intravascular growths to be broken off and carried away. In either case, let us see how dissemination could occur. The close proximity of the internal mammary vein would lead us to suspect it as being the principal means of dissemination. It is possible, also, that some of the intercostals would take part. In the venous circulation, the cells would, of course, be sent to the right ventricle of the heart, thence to the lungs. The right lung stands the greater chance of infection, since the right pulmonary artery is more nearly in line with the direction of the current sent out from the cardiac ventricle. Being too large to pass through the pulmonary capillaries, the emboli are arrested in the lung. Thus secondary foci are brought about, which are identical in structure with the original tumor. Inasmuch as this tumor has considerable cartilagenous tissue, and is not excessively vascular, I am inclined to the belief that metastatic deposits have not yet taken place. In case they have, we may look later on for the symptoms of pain in the lungs (more probably the right), dyspnœa, diminished or absent respiratory murmur, etc. It is quite probable, however, that if secondary foci have started up in the lungs, the tumor had so infiltrated itself in the surrounding tissues that a complete removal was not made. Under such circumstances we would have a springing up of the growth at the original source, and more symptoms referred to this source than to the secondary growth in the lungs.

The patient having recovered from the operation, the next question that concerns us is: Will the growth recur? This depends simply upon the complete removal of the original tumor. As sarcomas have a burrowing tendency, it is sometimes very difficult to remove all the malignant tissues. In this case portions of the fourth and fifth ribs were excised so as to be sure to take away any sarcomatous tissues that may have infiltrated itself therein. The fact that there is cartilage in this tumor is favorable to a late recurrence, if it recur at all.

In order of malignancy, sarcomas are arranged as follows:

1. Lympho-sarcoma.
2. Small, round cell sarcoma.
3. Melanotic sarcoma.
4. Spindle cell sarcoma.
5. Spindle cell sarcoma with islets of cartilage.
6. Myeloid sarcoma.

Even if it recur, dissemination is delayed and life prolonged.

Summing up the advantages of an operation, Bland Sutton* says: "It is, however, important to keep well in mind the fact that an operation, even if it does not cure, or even if it retards the progress of the disease, very often relieves the patient not merely of an encumbrance, but of a condition which is the source of great distress, mental anguish, and oft times intense pain."

NOTE.—Contrary to our expectations, the growth recurred, and our patient returned to the Sealy Hospital in July for further treatment. The neoplasm had recurred practically all around the line of previous excision, involving the sternal and axillary ends of the fourth, fifth and sixth ribs.

Professor William Keiller did the operation, he having charge of the surgical ward during Professor Thompson's absence. The growth was removed, both internal mammary arteries having been ligated so as to limit oozing from the sternum, a part of which had to be removed. The heart and pericardium were free, and pleura opened at one place. Wound healed by first intention, except a small place at axilla, where a temporary drain had been made. On leaving, two weeks afterwards, there was some tenderness and thickening over the third rib, which led one to suspect a recurrence at that place.

Patient returned to her home at Henderson, Texas. Inquiring as to her condition, the writer has been informed that the growth has recurred and is growing quite rapidly, but not yet associated with much pain. In the excision of these growths one should not forget the all important principle: *Remove all the malignant tissue, even at the expense of some healthy tissue.*

---

* Tumors, Innocent and Malignant.

## Figures and Tables

**Figure f1:**
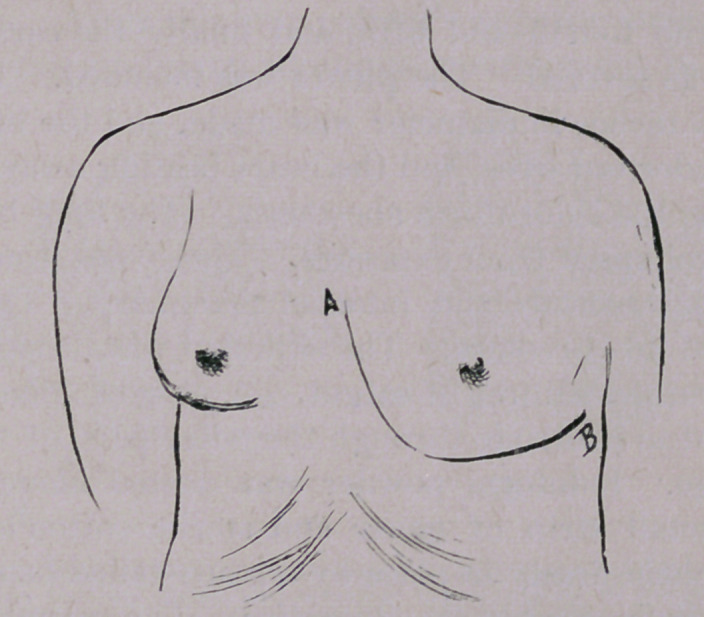


**Fig. II. f2:**